# Self-castration Resorted to as a Remedy for Seminal Emissions

**Published:** 1888-02

**Authors:** K. H. Boland

**Affiliations:** Atlanta, Ga.


### SELF-CASTRATION RESORTED TO AS A REMEDY FOR SEMINAL EMISSIONS.

REPORTED BY K. H. BOLAND, M. D., ATLANTA, GA.

In the summer of 1887 Mr. A. came to our office and requested me to dress a wound for him. On asking him as to the location of the wound, he stated that he had "just cut his testicles off and wanted the place dressed."

As a matter of course I was incredulous until I took him to the operating room and found on examination that sure enough he had completely emasculated himself so far as his testicles were concerned.

He had gone deliberately to work and shaved off the hair from the scrotum, tied his shirts up out of the way, tied his penis up to his belly, and with a coarse twine confined the testicles in the lower part of the scrotum, and with some more of this twine he formed a ligature around the scrotum higher up with a view to stopping hemorrhage, and then with one fell sweep of a keen razor he divided the scrotum between the two strings.

He used an old shirt to absorb the blood, of which there could not have been more than two ounces, and then the hemorrhage had ceased. He showed no other effect from the operation than a little weakness, though he had walked two squares after he had done the work.

With the aid of Dr. Renouff, my partner, I dressed the wound, first removing the clotted blood, after which I applied freely Prof. Gaston's antiseptic remedy (turpentine and camphor) and united the edges with some ten stitches on the median line.

The wound did not present a handsome appearance, as the center was depressed and the extremities stood out prominently in the form of a crescent. We dressed the wound with boracic acid and absorbent cotton, which was changed daily for ten days, at the end of which time perfect union had taken place except about half an inch in the centre from which there was a slight discharge.

There was no swelling nor extravasation of blood, and he suffered no pain or inconvenience from first to last, and the greatest trouble we had with him was to keep him from walking about, as he said he "needed exercise."

At first I doubted this man's sanity, but he talked and acted rationally in all other respects so far as I could see.

He assigned as a reason for this act that he had suffered for many years from seminal emissions, which had become the bane of his life, and so much did this seem to affect him that almost every day when he came to have the wound dressed he would ask if we thought there was any danger of the emissions recurring. He stated that on two former occasions he had tried to remove the offending members, and the second time he tried it he almost bled to death.

As a souvenir of the event he presented me with the testes, which I have preserved in alcohol. He stated he had never married, but in early life he had fallen into the habit of masturbation, and had tried many of the advertised remedies for "lost manhood,"

and assured us that for several years he had not slept in a bed, but had lain on the floor in order to avert these emissions by keeping cool.

After reporting to us for ten days he left for his home in a distant State.

Some three months after he left I received a letter from him in which he states that he has not gained much strength, but attributes his failure in this respect to malarial influence from which he has suffered; he states though he looks better and has gained in flesh, and in referring to his trouble he says he has had no more emissions; but from the manner in which he reverts to this it is evident that he is not certain but they may occur again.

